# Outcomes following persistent atrial fibrillation ablation using localized sources identified with Ripple map

**DOI:** 10.1111/jce.14092

**Published:** 2019-08-07

**Authors:** Daniel P. Melby, Charles Gornick, Raed Abdelhadi, Jay Sengupta, Manju Pai, John S. Zakaib, JoEllyn Moore, David G. Benditt

**Affiliations:** ^1^ Minneapolis Heart Institute and Foundation Minneapolis Minnesota; ^2^ University of Minnesota Medical School, Cardiovascular Division Minneapolis Minnesota

**Keywords:** atrial fibrillation, catheter ablation, outcomes, Ripple map

## Abstract

**Background:**

Ablation of persistent atrial fibrillation (AF) remains challenging. Identification and ablation of localized AF drivers may offer the possibility for improved outcomes. Ripple map is a novel software algorithm that may allow improved localization of possible AF drivers through the whole chamber graphical display of continuously recorded bipolar electrograms. The objective of this study was to determine whether regions of high‐frequency Ripple activation (HFRA) observed on Ripple map provide useful ablation targets in patients with persistent AF.

**Methods and Results:**

Consecutive patients underwent the first‐time ablation of persistent AF (n = 162) using a standard stepwise (n = 105) or a Ripple map guided approach (n = 57). Ripple map guided patients underwent pulmonary vein antral isolation followed by ablation of HFRA sites. Acute termination of AF was observed in 91.2% of the Ripple‐guided patients vs 52.4% in the stepwise approach, *P* < .0001. Following a single ablation procedure, after 18 months 98.2% of Ripple map guided patients were free of AF, compared with 81.4% of standard stepwise ablation (*P* = .005). Freedom from atrial tachycardia (54.4% Ripple map vs 52.4% standard, *P* = .9) or any atrial arrhythmia (52.6% Ripple map vs 39.0% standard, *P* = .10) did not differ between the two strategies. In a subset analysis (n = 30 of 56), Ripple map regions corresponded to sites with spatiotemporal dispersion in all atrial locations. No differences were observed in the rate of procedural complications.

**Conclusions:**

Ablation of HFRA sites identified with Ripple map resulted in a higher rate of acute termination and improved freedom from AF compared to a standard stepwise approach.

**Abbreviations**
AF: atrial fibrillation
AT: atrial tachycardia
HFRA: high‐frequency Ripple activity
LA: left atrial
NSR: normal sinus rhythm
PV: pulmonary vein
RA: right atrium

## 1 BACKGROUND

Pulmonary vein (PV) isolation remains the cornerstone of ablation for both paroxysmal and persistent atrial fibrillation (AF).^1^, ^2^ In paroxysmal AF, PV isolation can achieve approximately 80% 1‐year freedom from AF with optimal contact‐force radiofrequency or second‐generation cryoballoon ablation.^3^, ^4^, ^5^ In persistent AF, PV isolation alone has proven less successful likely due to the presence of non‐PV drivers.^6^, ^7^

Evidence in support of localized drivers maintaining AF include experimental^8^, ^9^ and clinical findings.^10^, ^11^, ^12^ Ablation of these sources with a procedural endpoint of AF termination may improve freedom from AF recurrence in both patients with persistent AF and the subset of patients with paroxysmal AF driven by non‐PV AF sources.^12^, ^13^

**Figure 1:**
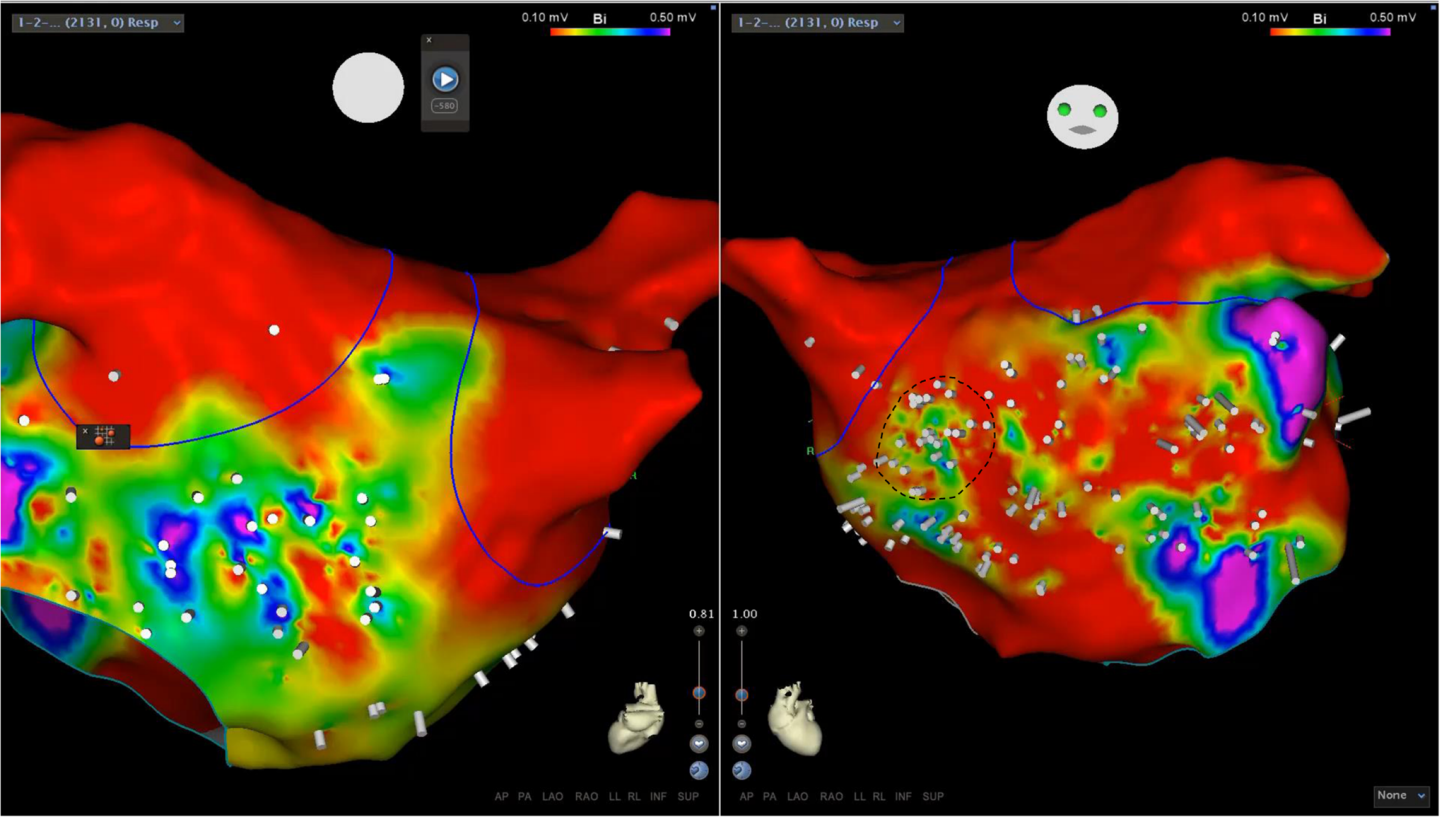
Example of Ripple Map. This map was obtained during sustained AF after PVI. Color shading represents bipolar voltage displayed on the anatomic map with a range of 0.1 mV (red) to 0.5 mV (purple). White Ripple bars representing the bipolar electrograms are shown perpendicular to the map surface with the size corresponding to the bipolar voltage. A total of 2131 points were acquired for this map with even distribution across the atrium. Some regions show no Ripple bars, indicating there was no depolarization at that location rather than lack of point acquisition. Analysis of this map demonstrated a HFRA site with continuous high‐frequency activation on the left atrial septum (dashed line). Ablation of this location resulted in AF termination to NSR. AF, atrial fibrillation; HFRA, high‐frequency Ripple activity; NSR, normal sinus rhythm; PVI, pulmonary vein isolation

Ripple map is a novel software feature of the Carto3 (Biosense Webster, Irvine CA) electroanatomic mapping system that graphically displays bipolar electrograms. (Figure 1) This visual marker displays a combination of depolarization frequency, electrogram fractionation, and voltage. The Ripple display corresponds directly to the recorded electrogram, with no interpolation between points or other processing. Using this methodology, it is possible to simultaneously display complete cardiac chamber electrograms recorded continuously over extended time intervals.

We hypothesized that (a) regions on the Ripple map displaying a concentration or cluster of high‐frequency Ripple activity (HFRA) would correspond to sites with spatiotemporal electrogram dispersion and (b) ablation of HFRA sites would improve the likelihood for intraprocedure AF termination and freedom from AF in patients undergoing an ablation procedure for persistent AF.

## 2 METHODS

This study was a single‐center retrospective evaluation of 162 consecutive patients who underwent first‐time ablation for persistent AF (continuous AF with a duration >7 days) from June 2013 to January 2018 using either a standard stepwise approach as developed by Haïssaguerre et al^14^, ^15^, ^16^ (n = 105) or a Ripple map guided approach (n = 57). Patients with very severely enlarged left atrial (LA), defined as a Carto measured volume greater than 300 cm^3^, were not included. Study participants were >18 years old undergoing ablation for AF. A minimum 90 days of follow‐up after the index ablation was required. There were no other exclusion criteria for this study including duration of persistent AF. Institutional Review Board approval was obtained, and all patients consented to research participation.

### 2.1 Ripple map guided protocol and mapping

Patients who were in normal sinus rhythm (NSR) at baseline underwent AF induction with atrial pacing from the coronary sinus. Before PV isolation, the PV and LA anatomy was constructed using a Pentaray catheter (Biosense Webster, 2‐6‐2 mm electrode spacing). Time continuous point acquisition was performed with the Pentaray catheter of the PV antrum and LA using the Confidense algorithm (Carto3; Biosense Webster). Electroanatomic point density was set at 1 mm to diminish regional point clustering variation based on catheter speed. Electrode stability was set at 3 mm to diminish movement artifact. Continuous electrogram recording was performed over a minimum of 10 minutes. At least 1500 bipolar electrogram points with equal electrogram density at all atrial locations was required to consider the map sufficient. A surface ECG lead that demonstrated a dominant R wave was used for reference annotation during point acquisition. No confidense filters were applied (specifically, local activation time, and cycle length stability).

After completion of the high‐density AF anatomic and point map, the Ripple map was viewed using a playback speed of 1 (this speed is an arbitrary parameter of the Ripple map program which determines the velocity of displayed electrograms; 1 is the slowest display speed whereas 5 is the fastest) A speed of 1 corresponds to approximately 1% of real‐time. A lowest displayed voltage of 0.03 mV and highest displayed voltage of 0.2 mV were used. The lower displayed voltage of 0.02 mV was necessary to correctly display regions of continuous low voltage electrograms. The upper boundary of 0.2 mV was chosen arbitrarily for map display, as the voltage was not used as a parameter to target for ablation. The Ripple window was prolonged to the maximum allowable time of 2.5 seconds to allow for a complete display of all acquired electrograms. HFRA was defined visually as regions of rapid and continuous Ripple activation. (Figure 1, Video S1) HFRA locations with the highest relative frequency by the visual analysis were targeted first, followed by progressively slower HFRA regions until termination. The highest frequency was defined specifically in relation to the atrium being mapped rather than an arbitrary absolute frequency. The largest anatomic regions as measured on the mapping system (typically greater than ~1.0 cm diameter) with HFRA were targeted for ablation first, followed by smaller regions. Isolated small segments, arbitrarily defined as less than 0.5 cm in diameter, were not ablated as these often represented isolated complex fractionated atrial electrogram (CFAI) electrograms.

Ablation proceeded with PV antral isolation followed by sequential ablation of the largest HFRA regions. Ablation of HFRA regions was performed in a cluster format and progressively expanded until local cycle length was observed to slow and there was loss of localized CFAE and dispersion. Ablation of HFRA sites was continued until AF termination occurred. HFRA sites not required for AF termination were not ablated as these sites did not appear critical for maintenance of AF. If AF remained after ablation of all the HFRA sites observed on the initial Ripple map, up to two additional Ripple maps of the LA were performed, followed by a Ripple map of the right atrium (RA). If AF persisted despite ablation of all recognized HFRA regions in either chamber or if the remaining HFRA region was adjacent to a critical structure (eg, the AV node or sinus node), cardioversion was performed to restore NSR. Linear ablation of the LA roof was performed for roof dependent macro reentrant atrial tachycardia (AT) or if extensive LA roof ablation was performed for HFRA in this location. Other linear or focal ablation was not performed unless a sustained AT was observed.

### 2.2 Comparison stepwise ablation protocol

A comparison historical cohort consisted of sequential patients who underwent the first‐time ablation for persistent AF using a standard stepwise approach as described by Haïssaguerre et al.^14^ For patients in the stepwise cohort, AF was induced with atrial pacing if NSR was present at baseline. Ablation was performed in the following sequence until AF termination occurred: PV antral isolation, posterior LA ablation, LA roof ablation, CFAE ablation, and lateral mitral isthmus ablation. In the CFAE step, ablation was performed for CFAE in both the LA and RA. If AF remained after the final step, cardioversion was performed to restore NSR.

### 2.3 All patients ablation protocols

All patients in both cohorts underwent radiofrequency ablation using an externally irrigated, contact‐force sensing catheter (SmartTouch and SmartTouch SF; Biosense Webster). A minimum of 5*g* contact was required before initiation of radiofrequency ablation. Power was fixed at 35 W and increased rarely to a maximum of 45 W if LA roof or lateral mitral isthmus block could not be achieved. All linear lines performed were tested for conduction block. PV entrance block was reconfirmed 30 minutes after initial isolation was observed. Esophageal temperature was monitored during posterior wall ablation. After the restoration of NSR through either ablation or cardioversion, an attempt at AF induction was performed with atrial pacing for up to 5 seconds in duration at the atrial effective refractory period or a minimum of 220 ms. Repeat ablation procedures for AF were performed using the same strategy as the first ablation (stepwise or Ripple guided).

The primary study outcome was 18‐month‐single‐procedure freedom from AF lasting longer than 30 seconds assessed by all available means including serial ECGs, Holter, extended outpatient monitoring, implanted device interrogation, and symptoms consistent with sustained tachyarrhythmia. Secondary outcomes included 18‐month‐single‐procedure freedom from AT, and any atrial arrhythmia, 18‐month freedom from AF, AT or any atrial arrhythmia after two procedures, acute AF termination during the index procedure, the use of antiarrhythmic medication, index procedure time, and complications.

### 2.4 Statistical analysis

Outcome analyses were performed for all patients who had a follow‐up. If both AF and AT occurred in the same patient during follow‐up, only one event was counted in the freedom from any atrial arrhythmia analysis. Survival analyses were performed to compare study groups for the primary and secondary time to event outcomes. Survival curves were generated using multivariable Cox proportional hazard modeling to adjust for baseline characteristics. For secondary categorical variables, the *χ* ^2^ test was performed with a statistical significance preset at 0.05. Continuous variables were compared using the Student *t* test.

## 3 RESULTS

### 3.1 Patients

The patient population comprised a total of 162 consecutive patients who underwent a first‐time ablation for persistent AF using either a Ripple map guided (n = 57) or standard stepwise (n = 105) approach. The standard stepwise approach was performed from June 2013 to November 2016. The Ripple map guided approach was performed from December 2016 to January 2018. Baseline demographics are listed in Table 1. There were more males in the standard‐map group (86% vs 67%, *P* = .009). No other significant differences were observed. The average duration of continuously persistent AF before the procedure was not different between the two groups (5.0 ± 5.7 months in the Ripple group vs 7.3 ± 10.8 months in the stepwise group, *P* = .14), nor was there any difference in the number of patients in continuous AF for more than 6 months (23% in the Ripple group vs 29% in the standard group, *P* = .18). There was no difference between in the groups in baseline left ventricular ejection fraction or LA size as measured by 2D echocardiogram.

**Table 1:** Characteristics of the patients at baseline

Characteristic	Ripple map (n = 57)	Standard stepwise (n = 105)	*P* value
Age (mean in y ± SD)	65 ± 10	65 ± 9	.90
Male sex, no. (%)	39 (67)	90 (86)	.009
Structural heart disease* (%)	8 (14)	18 (17)	.61
Left atrial diameter, mm	43 ± 5	45 ± 6	.062
Ejection fraction	53 ± 11	55 ± 11	.28
Duration since 1st AF diagnosis, mo	31 ± 41	41 ± 49	.17
Duration continuous AF before RFA, mo	5 ± 6	7 ± 11	.13
Constant AF for >6 mo	13 (23)	40 (29)	.18
Medical history, no. (%)			
Diabetes	9 (16)	22 (21)	.43
Hypertension	27 (47)	66 (63)	.06
Stroke or transient ischemic attack	3 (5)	7 (7)	.72
Vascular disease	16 (28)	27 (26)	.75
Renal disease	4 (7)	14 (13)	.22
Liver disease	0 (0)	3 (2.9)	.20
CHA_2_DS_2_‐VASc score	2 ± 1.5	2 ± 1.6	.78
Baseline medications, no. (%)			
BB or CCB	52 (91)	95 (91)	.88
Class Ic or III AAD	50 (88)	82 (78)	.13
Class Ic and III AAD	6 (11)	13 (12)	.73
Class Ic AAD	18 (32)	27 (26)	.43
Class III AAD	39 (68)	69 (66)	.73

*Note*: Plus‐minus values are mean ± SD. *P* < .05 was considered to indicate statistical significance.

Abbreviations: AF, atrial fibrillation; AAD, antiarrhythmic drug; BB, beta‐blocker; CCB, calcium channel blocker; RFA, radiofrequency ablation.

* SHD: congestive heart failure, constrictive pericarditis, amyloid cardiomyopathy, and valvular cardiac surgery.

### 3.2 Efficacy outcomes

After 18 months, significantly more patients in the Ripple map guided strategy were free of AF, compared to the standard stepwise strategy (98.2% vs 81.9, *P* = .005). (Table 2) This difference remained after adjusting for sex. There was no difference in freedom from AT (54.4% vs 52.4%, *P* = .9) or freedom from any atrial arrhythmia (52.6 vs 39.0, *P* = .34). After an average of 1.4 ablation procedures, freedom from both AF (98.2% vs 88.6%, *P* = .03) and any atrial arrhythmia (84.2 vs 69.5%, *P* = .04) were significantly higher in the Ripple group compared to the standard group. There was no difference in freedom from ATs after two ablation procedures (86.0% Ripple vs 76.2% standard, *P* = .15).

**Table 2:** Efficacy outcomes and follow‐up

	Ripple map (n = 57)	Standard ablation (n = 105)	*P* value
Outcomes, freedom from documented arrhythmia, no. (%)			
AF after one procedure	56 (98.2)	86 (81.9)	.005
AT/AFL after one procedure	31 (54.4)	55 (52.4)	.73
Any atrial arrhythmia after one procedure	30 (52.6)	41 (39.0)	.34
AF after two procedures	56 (98.2)	93 (88.6)	.03
AT/AFL after two procedures	49 (86.0)	80 (76.2)	.14
Any atrial arrhythmia after two procedures	48 (84.2)	73 (69.5)	.04
Follow‐up			
Follow‐up duration, d	462 ± 142	818 ± 448	<.0001
Cardioversion within 90 d index ablation	12 (21.1)	35 (33.3)	.10
Mean time to first documented recurrence, d	142 ± 108	209 ± 144	.06
Number of serial ECGs post index ablation	9 ± 6	10 ± 7	.85
Outpatient monitor or device check postablation	22 (38.6)	46 (43.8)	.52
AAD at last follow‐up	15 (26.3)	26 (24.8)	.83

*Note*: Ablation strategy based procedural efficacy and follow up results. Values are expressed as no. (%). Plus‐minus values are mean ± SD. *P* < .05 was considered to indicate statistical significance.

Abbreviations: AF, atrial fibrillation; AT, atrial tachycardia.

**Figure 2:**
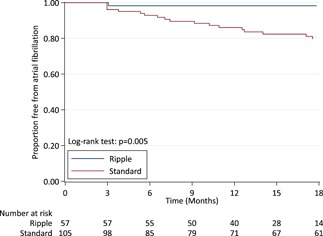
Freedom from atrial fibrillation. The graph shows Kaplan‐Meier estimates of freedom from documented atrial fibrillation after a single ablation procedure with or without the use of antiarrhythmic medications. There was a significant difference in survival free of atrial fibrillation in the Ripple map based strategy (98.2% vs 81.9%, *P* = .005)

**Figure 3:**
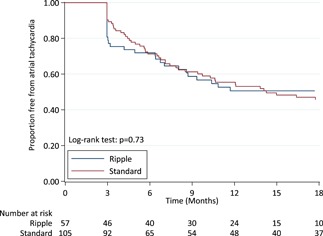
Freedom from atrial tachycardia. The graph shows Kaplan‐Meier estimates of freedom from documented atrial tachycardia or flutter after a single ablation procedure with or without the use of antiarrhythmic medications. There was no difference in survival

**Figure 4:**
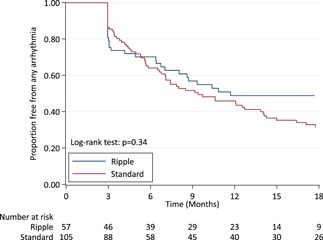
Freedom from any atrial arrhythmia. The graph shows Kaplan‐Meier estimates of freedom from any documented atrial arrhythmia after a single ablation procedure with or without the use of antiarrhythmic medications. There was no difference in survival

The survival curves for freedom from AF, AT, and any atrial arrhythmia are shown in Figures 2, 3, and 4. The survival curves were adjusted for sex due to its associations with the ablation approach; however, there was no evidence that sex confounded the relationship between ablation approach and freedom from arrhythmias.

### 3.3 Acute procedure characteristics and outcomes

Acute AF termination during the index ablation procedure was observed in 91.1% of Ripple map guided patients, vs 52.4% of standard stepwise patients (*P* = < .0001; Table 3) The mode of termination was not different between the two groups (21% to NSR and 67% to AT/atrial flutter (AFL) in the Ripple group vs 24% to NSR and 69% in the standard group). A mean of 4.3 ± 1.9 (range 1‐9) sites was identified with Ripple map and ablated. The most common regions identified on the Ripple map included the PV antrum in 100% of patients, LA roof in 73.7%, LA Septum in 70.2%, and inferior LA in 49.1%. HFRA was observed in the PV antrum of all patients, however, PV antral isolation alone only terminated 12.3%. The posterior LA was observed to have fast Ripple frequency in 49.1% but this region organized with PV antral isolation in 43% of this group and therefore posterior wall ablation was not needed. The RA and coronary sinus (CS) were mapped in 19.3% of patients when LA ablation failed to terminate AF. On subsequent maps, the previously ablated regions no longer exhibited HFRA in 89% of locations.

**Table 3:** Ablation procedural results

	Ripple map (n = 57)	Standard ablation (n = 105)	*P* value
Acute AF termination, no. (%)	52 (91.2)	55 (52.4)	<.0001
Mode of termination, no. (%)			
NSR	11 (21)	13 (24)	.76
AT/AFL	35 (67)	38 (69)	.84
MAT	6 (12)	4 (7)	.43
RF ablation time, min			
Total all sites	63.2 ± 17.4	59.2 ± 17.2	.17
HFRA sites	13.4 ± 10.3		
PV antrum and CTI	36.4 ± 9.0	36.3 ± 9.1	.78
AT/AFL/MAT	14.5 ± 6.5	15.1 † 7.2	.52
Initial mean LA pressure, mmHg	15 ± 4	15 ± 5	.60
Ablation sites for AF termination, no. (%)			
PV antral isolation	57 (100)	105 (100)	…
LA roof	42 (73.7)	95 (90.5)	.005
Posterior LA	16 (26.8)	91 (86.7)	<.0001
Inferior LA	28 (49.1)	32 (30.5)	.019
LA septum	40 (70.2)	69 (65.7)	.425
RA and CS	11 (19.3)	43 (40.9)	.005
CFAE	10 (17.5)	78 (74.3)	< .0001
Mitral linear ablation	27 (47.4)	57 (54.3)	.46
Procedural duration, h	2.9 ± 0.7	2.8 ± 0.7	.40
Fluoroscopy time, min	2 ± 2	6 ± 3	< .0001
AF present at baseline	43 (75.4)	81 (77.1)	.81

*Note*: Ablation procedural results. Mitral linear ablation was performed in the Ripple group only for sustained mitral isthmus dependent flutter. Inferior LA, Septal LA, RA and CS specific ablation data were also included with CFAE in the standard group. Values are expressed as no. (%). Plus‐minus values are mean ± SD. *P *<. 05 was considered to indicate statistical significance.

Abbreviations: AF, atrial fibrillation; AT, atrial tachycardia; CTI, cavotricuspid isthmus; HERA, high‐frequency Ripple activity; MAT, multifocal atrial tachycardia; PV, pulmonary vein; RA, right atrium; RF, radiofrequency.

In the Ripple map group, posterior wall ablation (26.8% vs 86.7%, *P* < .0001), LA roof ablation (73.7% vs 90.5%, *P* = .005), and CFAE ablation (17.5% vs 74.3%, *P* < .0001) were all performed less often. There was no difference in lateral mitral isthmus linear ablations. In the Ripple map group, mitral linear ablation was performed only for sustained mitral isthmus dependent flutter.

Total procedure RF time was not different between the two groups (63.2 ± 17.4 minutes in the Ripple group vs 59.2 ± 17.2 in the standard group, *P* = .17). Of this time, 13.4 ± 10.3 minutes were performed for HFRA sites in the Ripple group. Total ablation time includes all atrial arrhythmias (PVI, HFRA sites as well as all ATs, AFLs, and linear ablations) and is, therefore, longer than for ablation of AF alone.

**Figure 5:**
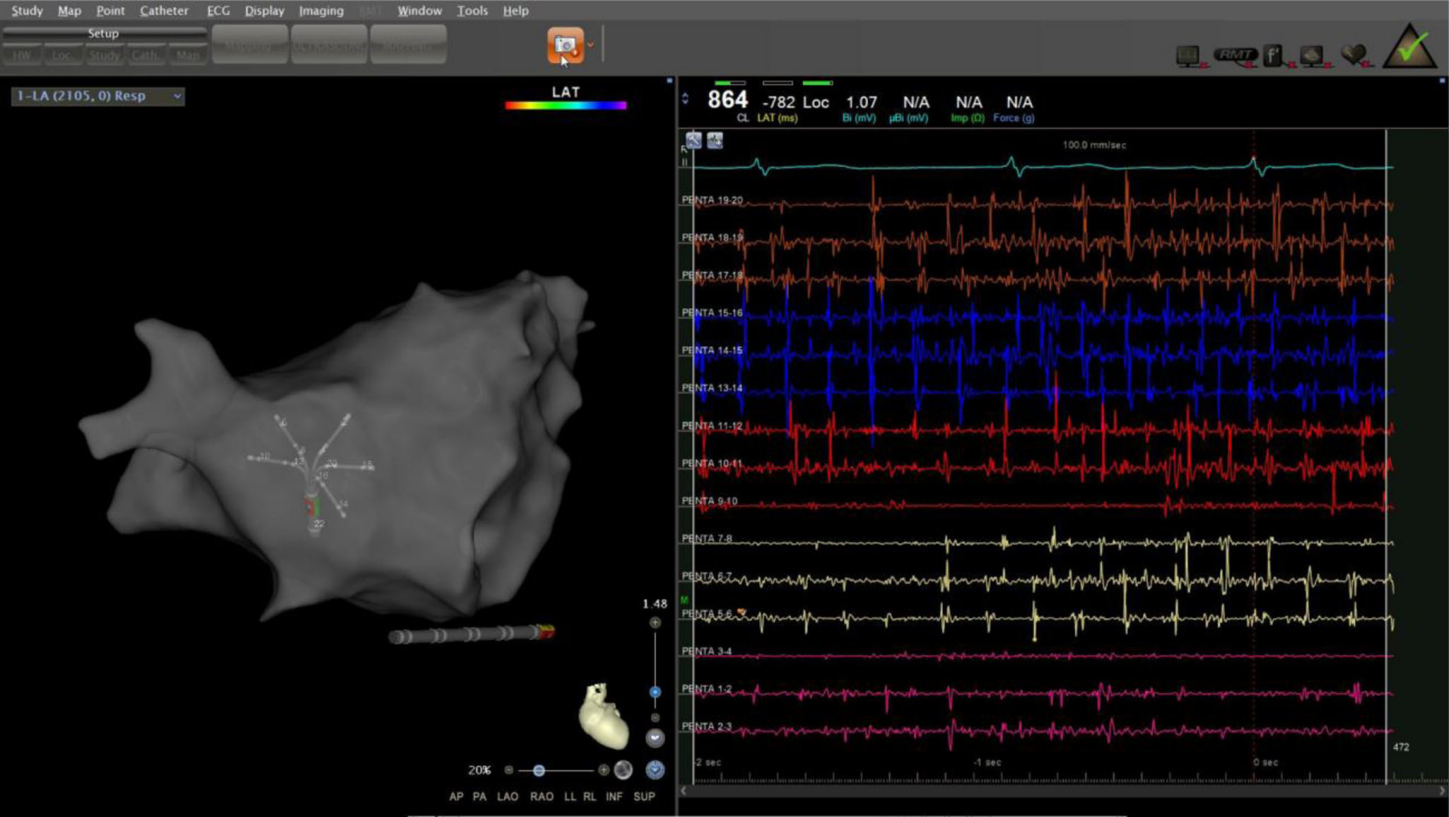
Rapid Ripple map site with spatiotemporal electrogram dispersion. Example of a Carto image with corresponding electrograms from the Pentaray catheter positioned at a region of HFRA. The Pentaray is located on the LA septum with widely spaced splines. At this location, there was high‐frequency Ripple activation and electrograms demonstrating CFAE and spatiotemporal electrogram dispersion. HFRA, high‐frequency ripple activation; LA, left atrial

A series of 30 patients in the Ripple group were evaluated by the proceduralist for electrogram characteristics in the regions with HFRA. In these 30 cases, a total of 142 HFRA sites were present. The proceduralist evaluated all Pentaray recordings available from the HFRA sites. A mean of five Pentaray recordings was present per HFRA location generating 50 electrograms for analysis. This number of Pentaray recordings allowed for adequate anatomic coverage of HFRA locations and was sufficient to characterize the HFRA location. The electrograms from these locations were designated as demonstrating spatiotemporal dispersion either in CFAE or sequential activation appearance as described by Seitz et al.^12^ In this evaluation group, all sites with HFRA had at least six electrograms (two electrograms from three separate Pentaray recordings) demonstrating spatiotemporal dispersion (Figure 5).

There were no serious procedural complications (transient ischemic attack, cerebrovascular accident, pericardial effusion, atrial‐esophageal fistula) within 30 days of the index ablation in either group.

## 4 DISCUSSION

In this retrospective analysis of Ripple‐guided ablation in patients with persistent AF who underwent ablation, the ablation of atrial regions visually determined to contain high‐frequency Ripple map activity (HFRA) resulted in (a) significantly higher rate of acute AF termination and (b) after an average of 1.4 procedures significantly higher freedom from AF and any atrial arrhythmia at up to 18 months follow‐up compared with a conventional stepwise ablation. In contrast, there were frequent occurrences of postablation atrial tachycardia with both strategies, and freedom from atrial tachycardia did not differ between the two groups.

An AF Ripple map for HFRA can be obtained during routine anatomic acquisition with the Pentaray mapping catheter. Rather than single electrogram analysis isolated in time, the Ripple map function allows the display of electrograms recorded continuously over extended time intervals throughout a cardiac chamber. Characteristics such as *d*v/*d*t, cycle length, timing, and voltage are visible. This combination allows for a potentially novel approach to the identification of AF drivers and can be displayed automatically on a 3D electroanatomic map. We did find high‐density recordings evenly distributed across the atrium were a prerequisite for accurate HFRA mapping. Further, in our experience in a subset of patients, electrogram analysis found regions of HFRA corresponded to regions of spatiotemporal dispersion. With Ripple map, the HFRA regions often had the appearance of CFAE, but also demonstrated electrograms with stepwise activation spatiotemporal dispersion as originally defined by Seitz et al.^12^ The work of Seitz et al^12^ previously demonstrated regional spatiotemporal electrogram dispersion (bipolar electrograms spanning most of the AF cycle length) is associated with the presence of AF drivers.

Similar to spatiotemporal electrogram dispersion or focal impulse and rotor mapping (FIRM) based mapping, HFRA for identification of localized AF sources offers possible advantages over isolated electrogram analysis such as CFAE. The Ripple map evaluation can be performed for complete RA or LA chambers allowing for regional comparisons of electrogram activation characteristics. In this study, we observed that this latter attribute was critically important for differentiating sites of possible AF drivers vs passive locations.

This study supports the pioneering work of other investigators including Narayan et al,^11^ Seitz et al,^12^ and Nadamanee et al^17^ who previously demonstrated a high rate of freedom from AF through ablation of identified AF drivers through alternative but supporting techniques. As was the case with these authors, we found an electrogram approach to mapping and ablation of AF drivers resulted in a higher rate of freedom from AF than a conventional anatomic approach. Our study differed from these prior investigations in so far as we only evaluated persistent AF rather than a mixed cohort of paroxysmal and persistent AF.

## 5 LIMITATIONS

This study is limited by its single‐center retrospective nature. Freedom from AF and AT is likely overestimated as systematic extended monitoring was not performed in all patients to reveal asymptotic paroxysmal tachycardias. The identification of critical HFRA sites are not strictly objective or quantified in this study methodology, therefore training is necessary to reduce observation variability among operators. This is not substantially different though from other methods such as spatiotemporal dispersion upon which these findings corroborate. Larger, randomized multicenter investigations are necessary to confirm the effectiveness of HFRA ablation.

## 6 CONCLUSION

In this study, we evaluated a novel ablation target for persistent AF. Ablation of sites which we have termed HFRA resulted in frequent termination of persistent AF and a higher rate of single‐procedure freedom from AF than was observed in a historical cohort in which a conventional sequential ablation strategy was employed. This technique complements well the findings of other investigators and supports mapping AF drivers and performing ablation beyond the PV antrum for persistent AF.

## Supporting information

Supplemental Video 1. Example of Ripple map following PV antral isolation in a patient who presented in persistent AF of 4 months duration. HFRA was present at the posterior superior aspect of the left atrial septum. AF terminated to NSR with ablation of this location. AF was thereafter non‐inducible during pacing the coronary sinus at 200ms. (see video file #1). The Ripple window for each acquired point was 2.5 seconds. A playback speed of 1 was used which corresponds approximately to 1% of real time
